# Comprehensive ADM1 Extensions to Tackle Some Operational and Metabolic Aspects in Anaerobic Digestion

**DOI:** 10.3390/microorganisms10050948

**Published:** 2022-04-30

**Authors:** Andrés Donoso-Bravo, María Constanza Sadino-Riquelme, Emky Valdebenito-Rolack, David Paulet, Daniel Gómez, Felipe Hansen

**Affiliations:** 1 ProCycla SpA, Camino Fundo El Junco SN, Melipilla 9580000, Chile; csadino@procycla.com (M.C.S.-R.); evaldebenito@aroma.global (E.V.-R.); dpaulet@procycla.com (D.P.); dgomez@procycla.com (D.G.); fhansen@procycla.com (F.H.); 2 ProCycla SL, Carretera Pont de Vilomara 140, 2-1, 08241 Manresa, Spain; 3 Department of Chemical Engineering, Universidad Técnica Federico Santa Maria, Valparaíso 2390123, Chile; 4 Aroma SpA, Camino Fundo El Junco SN, Melipilla 9580000, Chile

**Keywords:** anaerobic digestion, biogas, modelling, ADM1, semi-continuous fed, cellular transport, cell adaptation, working volume

## Abstract

Modelling in anaerobic digestion will play a crucial role as a tool for smart monitoring and supervision of the process performance and stability. By far, the Anaerobic Digestion Model No. 1 (ADM1) has been the most recognized and exploited model to represent this process. This study aims to propose simple extensions for the ADM1 model to tackle some overlooked operational and metabolic aspects. Extensions for the discontinuous feeding process, the reduction of the active working volume, the transport of the soluble compound from the bulk to the cell interior, and biomass acclimation are presented in this study. The model extensions are included by a change in the mass balance of the process in batch and continuous operation, the incorporation of a transfer equation governed by the gradient between the extra- and intra- cellular concentration, and a saturation-type function where the time has an explicit influence on the kinetic parameters, respectively. By adding minimal complexity to the existing ADM1, the incorporation of these phenomena may help to understand some underlying process issues that remain unexplained by the current model structure, broadening the scope of the model for control and monitoring industrial applications.

## 1. Introduction

Anaerobic digestion (AD) is expected to play a crucial role in tackling climate change and assisting in the transition toward a circular economy [1]. Modelling is a powerful tool that allows us to gain valuable insight about processes and, when properly applied, it can be useful for control, monitoring, and optimization purposes. The first mathematical representations of anaerobic digestion were created several decades ago. A significant milestone took place in 2002 when the International Water Association (IWA) came up with the Anaerobic Digestion Model No. 1 (ADM1) [2]. From that moment on, most of the published models have used the ADM1 as a basis, with the addition of different modifications, in order to represent some specific reactions. Modifications and extensions to the ADM1 model have been proposed to include processes, such as lactic acid generation [3], sulphate reduction [4,5], ethanol degradation [6], and cyanide degradation [7], among others. Thus far, the ADM1 is the most recognized model in anaerobic digestion [8]. After more than 20 years of application of the ADM1, and as stated by Weinrich and Nelles [9], normally, the same set of parameters are usually estimated during model calibration. Hence, a well-documented range of values for all the model parameters is available. The rest of the parameters have been seldom calibrated, thus keeping the original values proposed in the ADM1 report is good enough. In fact, one of the most challenging hurdles is to have a reliable substrate characterization for suitable model application [10].

Regardless of the model complexity, modelling, as such, is an abstraction of the reality, since several assumptions, considerations, and simplifications are included. In fact, even the ADM1 model can be considered as a parametric gray-box approach [11]. These assumptions can be related to operational conditions of the digesters, such as constant temperature, fixed pressure, and mixing quality, or some metabolic aspects, such as the disregard or bypassing of certain processes at a metabolic level. For instance, one of the most common assumptions is the homogeneity of the reaction media due to perfect mixing. However, and knowing that this assumption is hardly accomplished, plenty of research has been performed to tackle this issue by presenting new approaches based on ordinary differential equations [12,13] or using computational fluid dynamics (CFD) with partial differential equations [14]. CFD is expected to play an important role in AD modelling [15]. However, its computational cost still restricts its application mainly for reactor design since it is not viable to use it for monitoring and control. 

This study aims to analyze some aspects that have not been considered in AD modelling and to evaluate their incorporation into the ADM1 model in a simple way. Two operational aspects, namely the feeding strategy and the working volume reduction, and two metabolic aspects, the extra- intra- cellular transport and the adaptation, are tackled with new extensions of the ADM1 model proposed in this study. We aim at broadening the scope of the model by adding minimal complexity to the existing common model base.

## 2. The Rationale Behind

Four model extensions are proposed, two regarding operational aspects and two regarding metabolic aspects. Before describing the model equations, we provide theoretical background and the rationale behind the extensions that are proposed in the present study. 

### 2.1. Operational Aspects

#### 2.1.1. Feeding Strategy

For modelling purposes, a typical assumption is that the digester is continuously fed, which does not occur in reality. A continuous feeding rate is suggested to avoid process imbalances in digester operation [16]. Most full-scale reactors claim to possess a continuous process. However, to our knowledge, few of them are actually fed in a real continuous flow. Rather, they work in a semi-continuous fashion [17]. In other words, a certain amount of substrate, during a specific period of time, is fed continuously, but no real continuous feeding takes place mainly due to operational limitations, such as waste storage and availability, pumping system requirements, or substrate characteristics. Nevertheless, for any data processing analysis, it is considered that the reactor operates in continuous mode, especially for modelling applications, but also for global data analysis. The main reason supporting this assumption is that residence time is so big that the discontinuities will not exert a relevant effect on the bioreactor performance analysis. However, no real assessment of the impact of the feeding mode on the digester performance under a model-based analysis has been performed so far. In fact, few studies in the literature can be found where this pulse feeding mode has been considered in the model application. In the work of Mairet et al. [18], the discontinuous feeding of a lab-scale digester fed with microalgae was explicitly considered in the modelling application.

#### 2.1.2. Working Volume Reduction

Along with the substrate, particularly with semi-solid waste, such as sludge or manure, inorganic materials, such as sand and grit, will enter the digester tank and accumulate due to sedimentation at the bottom or in dead zones where the agitation system is less effective. This buildup will lead to the need for maintenance and the cleanup of the tank. This should be somehow monitored to anticipate the moment it will need to be carried out [19]. Furthermore, other compounds that are formed in the digestion process will precipitate and will not be removed in the digestate. In the end, the accumulation of material will lead to a reduction of the working volume of the digester or, in other words, an increase of the dead volume. This layer of dirt, which cannot be digested by the biomass, will cut down on the usable capacity of the digester [20]. Modelling-wise, the precipitation of material, mainly as a result of struvite formation and the interaction between sulphur and iron, has recently been a subject of study. The incorporation of these reactions leads to an increment of the model complexity due to the multiple reactions that have to be considered to represent these phenomena [21,22]. However, to the best of our knowledge, there have not been research studies trying to model the reduction of the working volume using conventional ordinary differential equations approaches (ODE) with the ADM1.

### 2.2. Metabolic Aspects

#### 2.2.1. Extra- Intra- Cellular Transport

The ADM1 model considers that any change in substrate composition will have an immediate impact on the growth rate of the microorganisms. Specifically, any change of soluble substrate will have an immediate influence on the biomass kinetics. This is clearly seen by looking at the expression of the growth rates which are based on the Monod kinetic. Of course, the instantaneous effect can be partially smoothed depending on the values of the affinity constant and the maximum substrate consumption rate. Likewise, the same instantaneous effect will take place for substrate inhibition. It is known that the cell membrane is impermeable to water-soluble compounds, such as glucose, amino acids, and LCFA, which means that a transport mechanism takes place inside the cell, and it has its own kinetics. Once inside the cell, these molecules enter their catabolic pathways. The transport will need time that, compared to the traditional modelling, will lead to a certain delay of the biomass response. This delay has been studied in other microorganisms such as microalgae [23]. The importance of the intracellular microbial activity in AD has recently begun to be the subject of research. Weinrich et al. [11] presented a strategy to couple the ADM1 with flux balance analysis, a method to predict metabolic function from genomic information. 

#### 2.2.2. Adaptation

Microbial adaptation has been a widely studied topic in AD because the capacity of microorganisms to adapt will determine the robustness of the process performance. Adaptation can be related to a change in the abundance of specific microbial species (selection of a genetically distinct population of microbes), population shift or change in dominance, or a mutation that results from changes in the activity of an enzyme that will provide a selective advantage by allowing growth under new conditions [24,25]. Particularly in AD, microbial adaptation usually occurs, in time, when the biomass is subjected to a new environmental condition, which can take place during digester start-up [26], new substrate incorporation or temperature change [27], the presence of trace elements [25], or changes in the concentration of compounds such as ammonia [28]. The current ADM1 model setting does not permit the representation of microbial adaptation/acclimation. The application of modelling to represent the adaptation phenomena in AD has been scarce. The use of batch tests with different substrate conditions to estimate some kinetic parameters related to biogas production, e.g., at different ammonia concentrations, has been a common approach to assessing adaptation [29]. In continuous operation, which is closer to how most digesters operate, to the best of our knowledge, only the approach presented by Kovalovszki et al. [30] incorporates a time-dependent function to account for the microorganism adaptation to temperature changes.

## 3. Methodology

### 3.1. Model Extensions

The base for the model extensions is the ADM1 developed by Batstone et al. [2] together with the parameters and model modifications proposed by Rosen and Jeppsson [31]. Furthermore, the role of the disintegration constant is minimized, as has been suggested by Batstone et al. [15], due to all the identification and interdependency disadvantages of the hydrolysis/disintegration two-step approach. Therefore, the composite concentration (X_c_) is set to zero so that the particulate material will enter the hydrolysis reaction directly partitioned as carbohydrates, proteins, and lipids. The hydrolysis and the disintegration constant were set at 0.5 d^−1^. The latter remains active only for the transformation of the biomass that has gone through decay. The rest of the parameters were set equal to the values presented by Rosen and Jeppsson [31].

#### 3.1.1. Feeding Strategy

The feeding strategy extension does not entail a modification of the model itself, but instead implies that the algorithm that solves the equations is divided in two operation modes working in a cycle: a batch reactor when no feeding is occurring and a continuous reactor when the feeding is taking place. The final conditions of a batch period correspond to the initial conditions of a continuous one, and vice versa. The mass balance for a substrate S in continuous and batch mode is shown, respectively, in Equations (1) and (2).

(1) $$\frac{dS}{dt}=\frac{F}{V}\left(S_{in}-S\right)\pm \sum r$$

(2) $$\frac{dS}{dt}=\pm \sum r$$

where F is the inlet flow of the digester, which switches to 0 in batch mode (no feeding taking place). The presence of the reaction rate(s) is the only term that always remains regardless of the operation mode.

#### 3.1.2. Working Volume Reduction

The working volume reduction comprises a modification of the mass balance for each state variable since the volume is no longer constant, so the classic mass balance of the chemostat needs to change. Because there is still an inlet and exit of media, the resultant balance is a mix between the balance for a fed-batch reactor and a continuous one. The general mass balance for a substrate S is presented in Equation (3) and the resolution of the derivative (chain rule) is shown in Equation (4).

(3) $$\frac{dSV}{dt}=F\left(S_{in}-S\right)\pm r\cdot V$$

(4) $$\frac{dS}{dt}V=F\left(S_{in}-S\right)\pm r\cdot V-\frac{dV}{dt}S$$

So, the question is, how does the working volume diminish over time? Here, we assume that the volume reduction is linear as a function of time (modelled by Equations (5) and (6), in its integral and differential form, respectively), with the slope (alpha, α) representing the accumulation rate of inorganic solids in m^3^ d^−1^ units. This parameter depends on the substrate characteristics, the mixing, and the pumping system, just like other apparent parameters such as the $k_{L}a$. $V_{0}$ is the initial working volume of the reactor at the beginning of the operation, which is defined as an initial condition of the state variable V.

(5) $$V=V_{0}-\alpha \cdot t$$

(6) $$\frac{dV}{dt}=-\alpha$$

The final balance can be written as it is presented in Equation (7), where only one new parameter is added to the model, the deposition rate alpha (α).

(7) $$\frac{dS}{dt}=\frac{F\left(S_{in}-S\right)}{V}+\frac{\alpha \cdot S}{V}\pm r$$

#### 3.1.3. Extra- Intra- Cellular Transport 

For the extra- and intracellular transport, three new state variables are introduced to the model. The soluble carbohydrates ($S_{su}$), proteins ($S_{aa}$), and lipids ($S_{lcfa}$) are now divided into two groups: those from the media bulk or extracellular and those inside the cell or intracellular. In this model extension, the microbial growth expressions depend on the internal soluble monomers’ concentration according to Equation (8) for the case of sugar consumers.

(8) $$\rho_{x}=k_{m.x\_su}\frac{S_{su\_in}}{K_{s}+S_{su\_in}}X_{su}$$

The expression that represents the rate of transfer between the bulk and inside the cell, given by Equation (9) for the case of sugar consumers, is similar to the one used to model liquid-gas transfer. This means that the phenomenon is governed by the gradient of the soluble monomer concentration between the exterior and the interior and a transport coefficient.

(9) $$TR\_S_{su}=k_{t\_su}\cdot \left(S_{su_{bulk}}-S_{su\_in}\right)$$

where $TR\_S_{su}$ is the transfer rate of soluble carbohydrates from the bulk to the interior of the cell, $S_{su_{bulk}}$ is the soluble concentration of carbohydrates in the bulk, and $S_{su\_in}$ is the soluble concentration of carbohydrates inside the cell. Again, only one new parameter was added to the model, $k_{t\_su}$, which corresponds to the transport coefficient (d^−1^).

#### 3.1.4. Adaptation 

In the case of the adaptation model extension, an explicit time-dependent function was added to the model. Considering that the adaptation process usually requires the microorganisms to develop a new enzymatic machinery, and as a first approach to the issue, the hydrolytic constant for the particulate carbohydrates was selected to be modified as the biomass adapts. Using Equation (10), the hydrolytic constant is now expressed as a function of time by a saturation-type equation.

(10) $$k_{h\_su}=k_{h\_su\_max}\cdot \frac{\left(t-t_{change}+1\right)}{K_{ad}+\left(t-t_{change}+1\right)}$$

where $k_{h\_su}$ is the hydrolytic coefficient function, $k_{h\_su\_max}$ is the hydrolytic coefficient (just like the one defined for the original ADM1), and $t_{change}$ corresponds to the moment of the operation where the change took place (in units of days, d). Only one new parameter was added to the model, $K_{ad}$, which is the adaptation coefficient (d). It is worth pointing out that other type of adaptations, for instance to higher ammonia concentrations would not lead to a change in the hydrolytic coefficient but rather in the inhibition coefficient to ammonia.

### 3.2. Modelling Conditions and Simulation

The model and the extension were implemented and simulated in Matlab 2021^®^ and the solver ode15s was used to solve the ODE system. A virtual digester of 330 m^3^ total volume (300 m^3^ working volume) operating at a hydraulic retention time (HRT) of 30 d (continuous flow of 10 m^3^ d^−1^) at mesophilic conditions (35 °C) was considered. The inlet characteristics of the substrate and the assessment conditions for each model extension are presented in Table 1. In order to minimize the effect of the initial conditions of the state variables, the final values of the state variables of a long-term simulation (over 100 d) were used as the initial conditions to assess the model’s extensions. The analysis of the simulations was focused on three variables, biogas production, methane content, and pH, taking into account that those variables are the most commonly monitored, by online measurements, and used in modelling applications for calibration and validation purposes.

## 4. Results

### 4.1. Feeding Strategy

**Figure 1:**
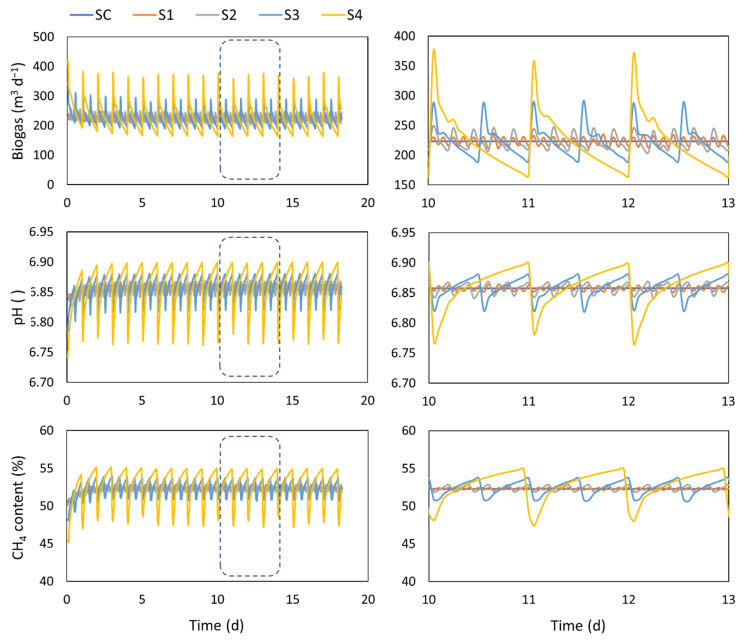
Performance of a digester under different feeding strategies. **Left**: results of 18 days of operation. **Right**: zoom in on only 3 days of operation.

The biogas production, the biogas composition, and the pH of the digestate for 18 days of operation for each of the assessed feeding strategies are shown in Figure 1. The second column of the figure represents a zoom-in on three operation days in order to afford a more precise consideration of the results. Simulations show that as the feeding becomes more spaced out over time, the biogas production loses smoothness, showing a high peak right after the feeding. Similar biogas production profiles results were also obtained by Bonk et al. [32]. The biogas flow reached peaks almost two-fold the average values moments after the feeding stopped with a once-a-day feeding (S4). Likewise, right before the next feeding takes place, the biogas flow is around 50% below the average. These variations can have an impact on the digester instrumentation maintenance as well as downstream processes, such as biogas purification, upgrading, and storage, that must be taken into account when the plant is designed. Regarding the process, although spot sampling may return different values, overall, the average daily biogas flows are similar for all the feeding strategies. In any case, during peak periods, the accumulation of certain compounds, such as the VFAs, can occur and influence the digester balance. Therefore, having a regular feeding has been recommended to avoid process imbalances [16]. However, later studies have also demonstrated that discontinuous feeding, when using VFAs as substrate, can make the digestate more resilient since more robust and diversified methanogenic populations are obtained [32]. The methane content of the biogas shows a similar trend compared to the biogas flow, although the curve is smoother. The methane content translates in the heat capacity of the biogas. Thus, it will influence the thermal or electrical generation at the end of the biogas treatment line. The methane content behavior depends on the pH due to the shift in the dissociation equilibrium between CO_2_ and HCO_3_. Thus, more (or less) CO_2_ is dissolved in the liquid phase (depending on the pH value), causing a change in the CO_2_ and CH_4_ concentration

As observed, the methane content in general is below than the average expected values (60–65%), which is explained by the fact that, for this study, the original values of the ADM1 were used. According to some studies, the value of some coefficients related to the methane formation for carbon need to be adjusted higher [31,33].

The pH is less affected by the feeding strategy than the biogas flow, which is expected since the variables measured in the liquid media are normally more robust due to the influence of the working volume and the HRT of the process. Nevertheless, some pH variations are observed, but they do not reach values that could be considered dangerous for the digester operation since they remain close to neutrality (depending on the lower and upper limits for pH inhibition). 

### 4.2. Working Volume Reduction 

**Figure 2:**
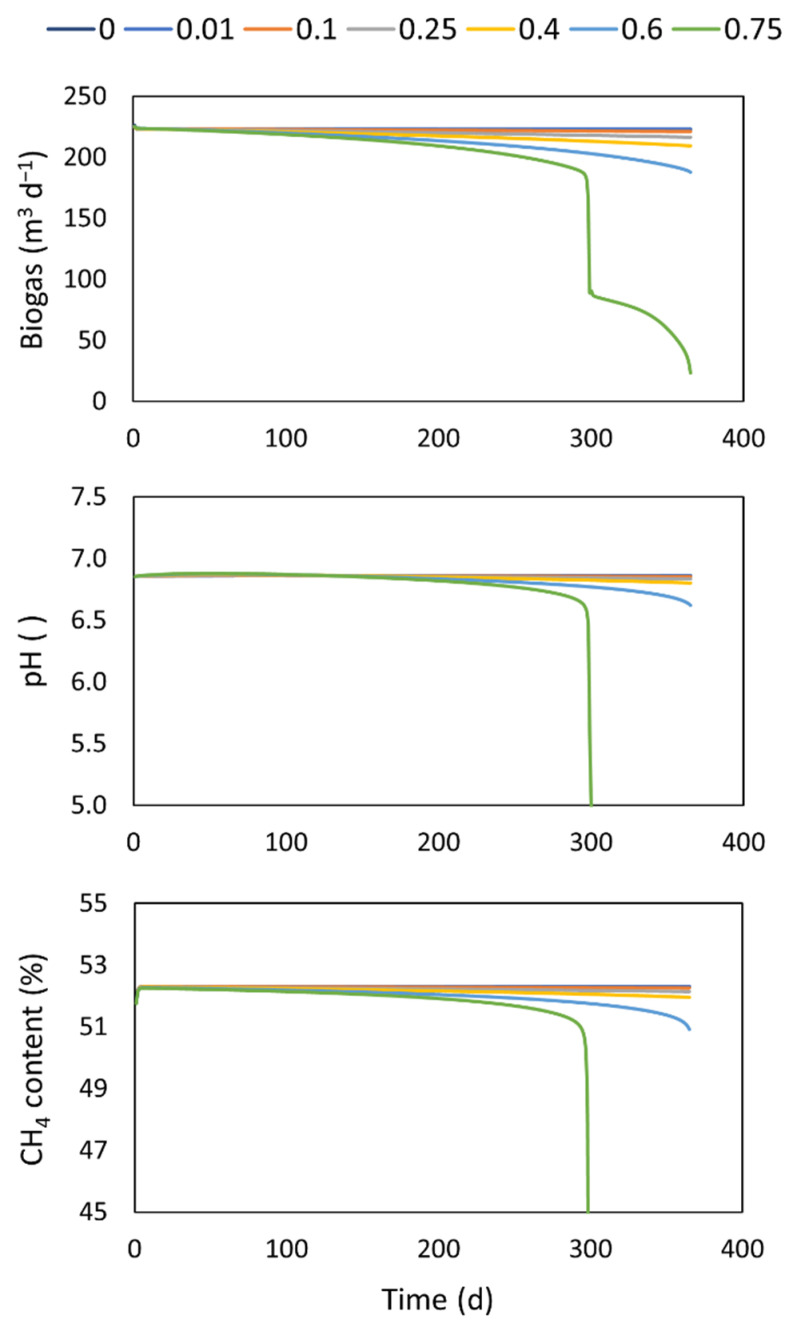
Performance of a digester under different rates of solid material accumulation.

The biogas production, composition, and the pH behavior in the digester, for a year of operation, at different accumulation rates of solid material (α), are shown in Figure 2. It is worth noting that, in this model extension, the digester does not represent a constant continuous reactor anymore (reactor volume is now a state variable, see Equation (6)). It can be seen that below values of 0.4 m^3^ d^−1^ of the accumulation rate of inert material, the impact on biogas production is low. Indeed, at this rate, the biogas decreases by 5% due to the loss of working volume. At a rate of 0.6 m^3^ d^−1^, the biogas production decreases 16% compared to the values when no working volume is lost (conventional continuous operation). At higher accumulation rate values, the reactor collapses with a sharp drop in the biogas production and pH associated with the washout of the methanogenic biomass. It is interesting to note that the effect of the working volume has, for a long period, a steady negative effect on the biogas production that steadily decreases due to the reduction of the biomass activity. However, the collapse of the system could occur in a matter of a few days. In this case, the system will need to be stopped and reinoculated completely with all the operational and waste management consequences that a situation such as this entails. By having a well-established model, this accumulation rate could be estimated from the biogas production and could be used to estimate the need for a clean-out and digester maintenance.

### 4.3. Extra- Intra- Cellular Transport 

**Figure 3:**
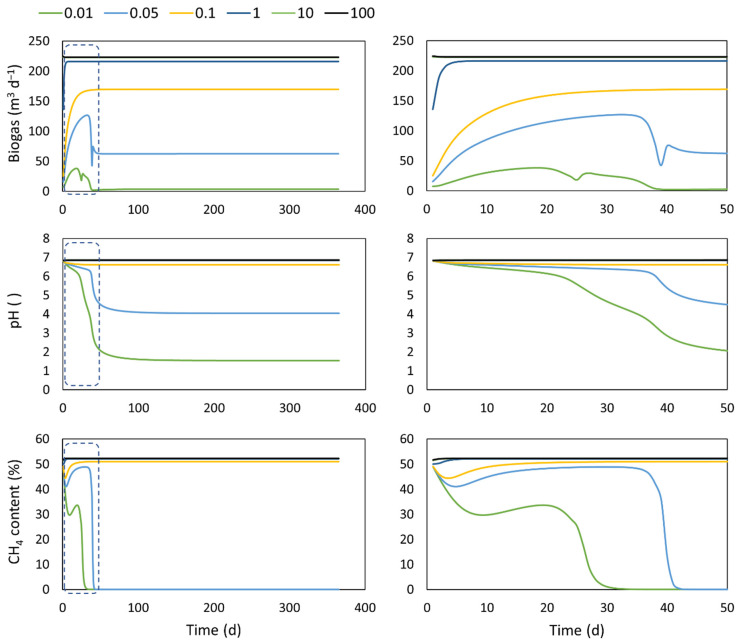
Performance of a digester under different extra- intra- cellular transport kinetics. **Left**: results of 365 days of operation. **Right**: zoom in on 50 days of operation.

The response of the biogas production flow, the biogas composition, and the pH of the digestate regarding the proposed extra- and intracellular transport kinetics extension is shown in Figure 3. At values of the transport coefficient above 1 d^−1^, the system operates without a limitation given by this transport process. This can be related to a system well adapted to the substrate, where the transport of monomers to the interior of the cell is a consolidated mechanism. Under these conditions, the concentration inside the cell is similar to the one in the bulk. Thus, similar biogas production values are obtained. At lower values of the transport coefficient, $k_{t}$, (i.e., 0.1 d^−1^), the measured variables begin to be limited by this extra- and intracellular transport process, and the system reaches a steady-state with lower biogas production accompanied by a modified abundance of the microbial species given by the steady-state substrate concentrations. Similar results were obtained for a culture of *Escherichia Coli* in a chemostat [34]. Lower values of this coefficient, which could be associated with a more recalcitrant substrate whose soluble fractions are not properly absorbed by the microorganisms inside the cell, led to the loss of the methanogenic activity due to the washout of the biomass, since they cannot keep up with the required microbial growth to offset the solid retention time. At 0.05 d^−1^, the biogas is mostly produced by no methanogenic biomass. Thus, it is composed mainly of CO_2_, whereas at values of 0.01 d^−1^, the biogas drops to almost zero, with a minimum generation of biogas made up of H_2_ and CO_2_. As the transport coefficient values decrease, the pH values tend to level off to lower values which are explained by the low generation of VFAs due to the transport restrictions. The fact that no extra- or intra- cellular transport is normally considered can lead to an overestimation of the digester performance as well as an anticipation of the system’s response since the delay related to this transport is not accounted for. This delay depends on the types of compounds that need to enter the cell. In this study, the same transport coefficient value was considered for all the monomers, which is probably not very accurate in a real case. For instance, the light required in a microalgae culture has a very fast transport kinetic, and thus a minimum delay is observed [23], whereas other compounds, such as ammonia or inorganic carbon, need a certain time to enter the cell, leading to a delay in the system response [23,34,35]. In general, recently, some modifications to the structure of the ADM1 to address some microbiological aspects of the model have been also proposed [36].

### 4.4. Adaptation 

**Figure 4:**
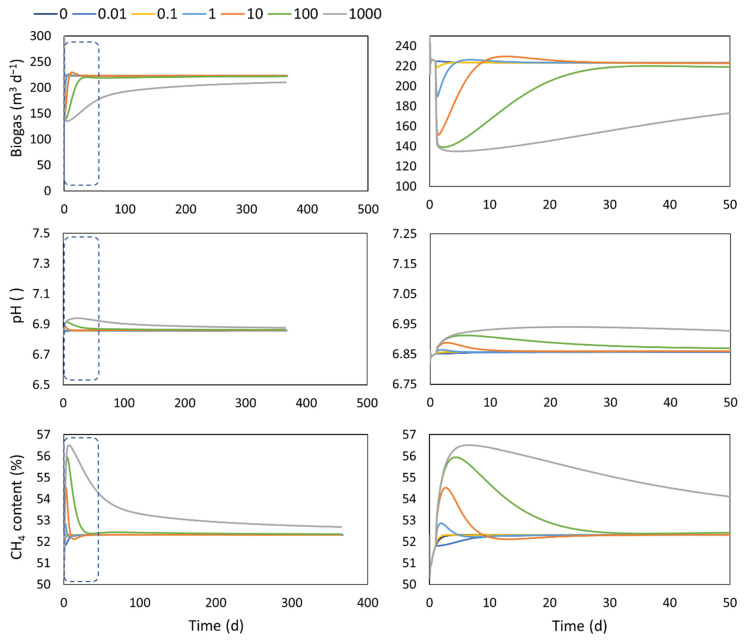
Performance of a digester during start-up with different inoculum characteristics. **Left**: results of a year of operation. **Right**: zoom in on the first 50 days of operation.

The extra- and intracellular transport can cover some aspects concerning the biomass adaptation response, but it still considers a fixed set of parameters. In this case, the adaptation extension of the model includes the dynamic evolution of the hydrolytic coefficient over time. In the incorporated function for this extension, the values of $K_{ad}$ are related to the characteristics and quality of the inoculum used to seed the digester. The performance of the digester’s startup in a period of one year and the zoomed-in 50-day period, using different values of $K_{ad}$, is shown in Figure 4. The model extension is able to represent the dynamic of the biogas production, during the startup, considering the quality of the inoculum represented by the $K_{ad}$ coefficient. The values of $K_{ad}$ will have an impact on the biogas production trajectories as well as the final biogas values that can be reached in a certain period of time (in the long run, it will closely approach the theoretical value due to the asymptotic form of the function). Taking into account a high $K_{ad}$ value, which can represent a poor-quality inoculum, the daily biogas production is constantly lower compared to the case where minimal adaptation is required (low $K_{ad}$ values). The main difference, as is observed in the zoom-in graph, is attained in the first two months of operation with 40% lower biogas production than the base case with minimum adaptation required.

With $K_{ad}$ values between 0.1 and 10 d, a period where the biogas production is higher than the base case can be observed (this is more evident in the case of $K_{ad}$ = 10 d, between day 8 and 20), after which similar values around 220 m^3^ d^−1^ are obtained. This behavior can be explained by a higher accumulation of VFAs as the time goes on and the adaptation process moves along, and it comes as a response to the previous period where the biogas values are notoriously below the base case where the process is strongly limited by the hydrolysis reaction. The fact that this extension affects the hydrolysis reaction leads to the methane content having the opposite behavior, as when *K_ad_* increases, the methane content also increases. This effect upon the hydrolysis reaction has a more important influence on CO_2_ production, which makes the biogas composition to behave this way. 

In general, the pH does not seem to be significantly affected in the representation of the adaptation process. It remains between recommended values to operate digesters.

**Figure 5:**
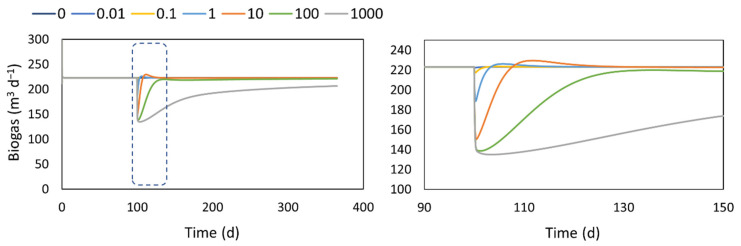
Performance of a digester during the adaptation to a co-substrate addition with different substrate characteristics. **Left**: results of a year of operation. **Right**: zoom in on the first 50 days of operation.

The simulated response of a digester when a new (co)substrate is added on day 100th is presented in Figure 5 (only the biogas production is shown, whereas the pH and the methane contents behave as shown in Figure 4). In this case, the values of $K_{ad}$ can be associated with the properties of the substrate with respect to the anaerobic biomass developed in the digester. The highest values are associated with a substrate that is more recalcitrant to the existing anaerobic biomass, while the low values represent a substrate with similar characteristics to the one that is currently fed to the system. Similar to the start-up results, the (co)substrate addition leads to a drop of the biogas production that is proportional to the magnitude of $K_{ad}$. However, the biogas production bounces back to the base case values, showing even higher values for short periods of time. 

Making the kinetic parameters or stoichiometric coefficients become variables of the model has been evaluated in a handful of studies. Overall, most of these studies have focused on the stoichiometry of the ADM1 representing the anaerobic digestion process. The catabolic yield for acetate and butyrate were defined as a function of the hydrogen concentration and the pH in the digester by Rodríguez et al. [37], although no significant differences were observed in the results compared to the standard fixed yield model. Similarly, the acidogenic stoichiometry of the ethanol fermentation was considered a variable in the work of Shi et al. [38], as well as for biohydrogen production by dark fermentation in the work of Penumathsa et al. [39]. Regarding the kinetic parameters, the hydrolytic constant has been transformed into a function of the temperature of the system by Donoso-Bravo et al. [40] and Kovalovszki et al. [30].

### 4.5. On Model Extension Validation

As one may think, these theoretical approaches presented here need to be validated with experimental results, particularly with the extensions where new parameters were incorporated. For the working volume reduction extension, the operation in a (semi) continuous mode of a sludge digester at the same HRT and OLR for long period of time can be used to calibrate the accumulation rate parameter. Under those conditions, one can expect that the only variable that can change the reactor performance is the reduction of working volume due to the accumulation of inert material. In the case of the extra- intra- cellular extension, the addition of a specific soluble substrate in a pulse manner can be carried out while observing the system response in terms of biogas flow and pH. This way the transport coefficient can be estimated depending on the observed delay between the system’s disturbance and the system’s reaction to it. For the adaptation extension, an experiment where a sudden change of the type of substrate used, without changing the total OLR and the HRT, could be used to estimate the adaptation coefficient.

## 5. Conclusions

Four new extensions to the ADM1 model have been proposed to cover two operational aspects, namely the feeding strategy and reduction of the working volume as a result of solid material accumulation, and two metabolic aspects, namely the transport of monomers from the bulk to the cell and the biomass adaptation to a substrate given the time of exposure. 

The feeding strategy can influence the curve of biogas production and, consequently, the quality sensors used to measure it, and it has an effect on the downstream process for biogas purification and storage. The average daily production is not affected as long as inhibitory levels of some intermediate variables are not exceeded. The reduction of the working volume of the reactor depends on the rate of material accumulation, which can be estimated from the biogas production data. This phenomenon leads to a reduction of the activity of the microorganism and eventually to a total collapse of the anaerobic digester. The extra- and intracellular transport kinetics, regarding the soluble compounds, considered in the model application allows us to account for the delay in the response of the system compared to a conventional instantaneous model kinetic. Variable kinetic parameters as a function of the time of exposure can be considered to obtain a more realistic picture of the adaptation that takes place during, e.g., the start-up of the digester or the addition or replacement of the substrate fed into the reactors.

## Figures and Tables

**Table 1:** Inlet conditions and assessed simulated operating conditions for the model extensions.

**Inlet Conditions**			
VS	30 kg m^−3^	Ammonia nitrogen	2 kg m^−3^
COD/VS ratio	1.5	Inorganic Carbon	0.004 kmole m^−3^
CODs/CODt ratio	0.1	VFAs	0 kg COD m^−3^
Carbohydrate’s fraction ^1^	0.4	Soluble gases	0 kg COD m^−3^
Protein’s fraction ^1^	0.4	Anions	0.04 kmole m^−3^
Lipid’s fraction ^1^	0.2	Cations	0.18 kmole m^−3^
Inert fraction	0.2		
**Model Extension: Feeding Strategy**			
**Strategy**	**Notation**	**Momentaneous Flow**	
Continuous	SC	10 m^3^ d^−1^	
10 blocks of 1.2 h feeding	S1	20 m^3^ d^−1^	
5 blocks of 1.2 h feeding	S2	40 m^3^ d^−1^	
2 blocks of 1.2 h feeding	S3	100 m^3^ d^−1^	
1 block of 1.2 h feeding	S4	200 m^3^ d^−1^	
**Model Extension: Working Volume Reduction**			
Inlet flow	10 m^3^ d^−1^	Accumulation rate α: 0 (base case), 0.01, 0.1, 0.25, 0.4, 0.6, 0.75 m^3^ d^−1^	
**Model Extension: Extra- Intra- Cellular Transport**			
Inlet flow	10 m^3^ d^−1^	Transport coefficient $k_{t}$: 0.01, 0.05, 0.1, 1, 10, 100 d^−1^	
**Model Extension: Adaptation**			
Inlet flow	10 m^3^ d^−1^	Digester start-up $t_{change}$ = 1 d New (co) substrate addition $t_{change}$ = 100 d Adaptation constant $K_{ad}$: 0 (base case), 0.01, 0.1, 1, 10, 100, 1000 d	

^1^ The fraction refers only to the biodegradable fraction. VS: Volatile Solid, CODt: total Chemical Oxygen Demand, CODs: soluble Chemical Oxygen Demand, VFAs: Volatile Fatty Acids.
